# A Review of Certain Experimental Findings upon the Accessory Food Factors

**Published:** 1921-06

**Authors:** John Albert Marshall


### A REVIEW OF CERTAIN EXPERIMENTAL FINDINGS UPON THE ACCESSORY FOOD FACTORS

BY JOHN ALBERT MARSHALL, M.S., D.D.S., PH.D.

One of the most interesting fields of research in physiological chemistry is the study of the so-called vitamines, or the "accessory food factors." As yet the behavior of these substances in the body, how they act, or what they are chemically, has not been clearly worked out, but it has been well established that their relation to the health of the individual is apparent and important. The first mention of this particular relation was made by Eijkman in 1897. He pointed out that eating of unpolished rice would prevent the development of the oriental disease known as beri-beri. In our California history we find many references to scurvy, a disease prevalent among the crew of sailing vessels. The outbreak occurred when conditions were such that only a diet of salt meat and hardtack was available. Later, it was observed that if fresh meat or vegetables, or even freshly baked bread, was added to the diet, that the disease would not recur. The underlying causes, however, which appeared to control the outbreak, as well as those which alleviate the symptoms, were not recognized until very recently. Within our own recollection, the Alaska gold rush excitement contains many references to the experiences of pioneers suffering from scurvy. The attempts at an explanation of this conditions were based on a research bearing on the properties of the different classes of foodstuffs.

Foods are classified in general into four main divisions:

First—The nitrogenous substances or the proteids which are found in meat, eggs, milk, and to a lesser extent in certain vegetables.

Second—The fats, both vegetable and animal fats as butter, olive oil, fat meat, etc.

Third—The carbohydrates as sugar and starches.

Finally, the inorganic substances which include water and the mineral salts.

The function of each of these are well known and it has been assumed in the past that if these different ingredients were present in the correct proportion that the individual could maintain a normal healthy existence. This is not true, for it has been determined that animals which are fed on these articles of diet in a pure form develop definite symptoms. In other words, if an animal is given proteid, fat, carbohydrates and mineral salts, all of which have been chemically purified, the weight, growth and general condition of the animal soon falls below normal.

Something is removed in the purification which is present in our natural food, a constituent which is quite necessary for the maintenance of life. Further than this, a diet of salt meat and hardtack is relatively deficient in these materials.

One of the pioneers in this field named these substances vitamines, because it was thought at first that they were closely related to a chemical group derived from ammonia, called amines. Since their presence is essential to the life and vitality of the organism, the words *vitality* and *amine* were combined to produce the hybrid word "vitamine." Although this term is now recognized as incorrect, it has the virtue, at least, that it is much shorter than the suggested one, "accessory food factor." It is important to understand at the outset that there are many materials in the animal body which are present in the most minute quantities, but which, if totally absent, produce profound clinical symptoms. The so-called "vitamines" are only one class of substances which fall in this category.

Lately, experiments have been reported which indicate that there is a close connection between the normal processes of calcification in the body and the presence or absence of these vitamines. One of the most interesting experiments, from the dental standpoint, is one which was reported from England. The experimenter, Mrs. Mellanby, first endeavored to trace the factors involved in the development of sound teeth and the growth of jaws; and, second, the influences which appear to affect erupted teeth and to bring about an established immunity to caries and other diseases. About 280 dogs were used in the research and many of the experiments were repeated so as to eliminate any possible errors which might arise in computations of variation in growth, etc.

Without going too deeply into the chemistry of these interesting food accessories, I merely want to point out that there are three separate vitamines known today:

First, the antiscorbutic, which controls the development of scurvy.

Second, the Water Soluble B, or antineuritic, the absence of which produces polyneuritis.

Finally, the Fat Soluble A. The term "Fat Soluble A" is intended to convey some idea of the physical properties of this group, namely: the relative solubility of these vitamines in fats. The Fat Soluble A compounds are found in many of the natural foods—for example, in butter, fish, oils, milk, eggs, cheese, cabbage, spinach, etc.

Experiments were performed not only on the very young bottle-fed puppies, but also upon the developing embryos. Defective teeth were produced when the animals were fed diets which contained lard, skimmed milk, white flour, rice, sugar, oatmeal, and some of the vegetable oils, diets which are deficient in vitamines. A notable difference was observed in the development of the teeth and jaws when the animals were fed on a diet which contained such foods as butter, animal fats, excepting lard, milk, eggs and even cod-liver oil. Not only was the deposition of lime increased with a diet which contained this Fat Soluble A, but the actual structure of the teeth and jaws was noticeably different. In those cases where the diet was deficient in Fat Soluble A, the following points were demonstrated:

First, an *abnormal growth* of the *jaws* and alveolar processes, the abnormality consisting in size and relation to the teeth.

Second, an *irregularity* of the teeth after eruption.

Third, a *delay* in the *eruption* of the dentition, also a delay in the *shedding* of deciduous teeth.

Fourth, the production of *enamel* which was *defective* structurally.

Fifth, the production of *dentin* which was *defective*, in that it showed large numbers of interglobular spaces; and, finally, a difference in the *rate of growth* in the animal.

It has been shown by Mrs. Mellanby that the younger the animal the more essential is Fat Soluble A, and further, that the more rapid the growth of the animal the greater is the quantity of this substance needed to maintain the organism in a healthy condition. These facts appear to be confirmed when we take a hasty survey of the present diet of the world. For instance, during the war many children were deprived of vitamines through conditions imposed by the restricted diets. In many instances it has been observed that the structure of the skeleton and of the teeth of individuals born and reared under these conditions is defective. Meat and animal fats did not play as important a part in the diet as the cereals. Butter was superseded by vegetable fats. Whole milk was excluded in some cases almost entirely. Deficient dietaries must necessarily have their effect upon growth, an effect which we can not fully estimate or measure at the present time. These facts are beyond the experimental stage and they indicate the necessity for the dentist of today of realizing the importance of diet.

The beginning of digestion is in the mouth, and not in the stomach. The salivary enzymes play an important part in digestion. They are controlled by the act of masticating. Efficient mastication can not be accomplished by an individual possessing unsound teeth. If there is any group of scientific men which should understand diet and digestion it is the group of dentists. The dentist restores the masticatory mechanism to normal function. He can better appreciate what that function is when he studies the problems of food and of the digestion and metabolism of food.

Other experiments have been performed, some of which have already been reported by Dr. Howe, and others not yet reported, which appear to trace a closer relation to the teeth than has been indicated. For instance, in experimental work which has been conducted in the George Williams Hooper Foundation for Medical Research, it has been possible to produce a condition which, from the clinical standpoint at least, possesses some characteristic symptoms in common with pyorrhea. The symptoms which have been observed upon laboratory animals are as follows:

At the onset of the attack the teeth become sore and gradually protrude from their sockets. The animal refuses food on account of this acute pericementitis. Following this, or, perhaps, coincident to it, there occurs a deposition of material about the necks of the teeth which closely resembles salivary calculus. This calculus accumulates rapidly and soon a severe gingivitis develops. In addition to this, a saliva is produced which contains a relatively large amount of mucin and which is of a most offensive odor. I have not been able to trace the actual formation of pus pockets about the teeth, but up to that point, at least, the sequence of events following a deficient diet approximates very closely the stages of our clinical pyorrhea. If the latter stages can be produced experimentally, it will reopen an old question, which in earlier dental history was as sure of producing a fight as the question today of extraction or conservation of pulpless teeth. Many men believe that G. V. Black settled the dispute when he stated that pyorrhea (chronic suppurative pericementitis) is a local disease rather than a local manifestation of a systemic disorder. It now appears that there is a possibility of a development of this latter viewpoint. For if the laboratory worker can produce the symptoms of pyorrhea by imposing a restricted feeding schedule, he will prove that the disease is a local manifestation of malnutrition and faulty diet.

The ultimate correction of this condition will come from two things:

First, a local prophylactic treatment or deep scaling.

Second, a carefully devised systemic treatment which will be based upon *his knowledge of foods.—Pacific Gazette.*

